# Preservation of periprosthetic bone with a collum-fixated short stem versus conventional metaphyseal-fixated stems in total hip arthroplasty: a randomized controlled trial with 2-year follow-up in 61 patients

**DOI:** 10.2340/17453674.2026.46481

**Published:** 2026-08-05

**Authors:** Anders TJØNNELAND, Janus D CHRISTIANSEN, Thomas JAKOBSEN, Poul T NIELSEN

**Affiliations:** Interdisciplinary Orthopaedics, Department of Orthopaedic surgery, Aalborg University Hospital, Aalborg, Denmark

## Abstract

**Background and purpose:**

We aimed to compare the effect of 2 femoral stem designs on periprosthetic bone mineral density (BMD) after total hip arthroplasty in a randomized controlled trial. We hypothesized that a short collum-fixated stem (Primoris) would better preserve proximal BMD than a conventional metaphyseal-fixated and diaphyseal engaging stem (Echo).

**Methods:**

61 patients with primary osteoarthritis were randomized to receive either the Primoris (n = 31) or Echo (n = 30) uncemented femoral stem. DXA scans were performed preoperatively, at 6 weeks, 12 months, and 24 months. The prespecified primary outcome was periprosthetic BMD at 24 months and patient-reported outcomes (EQ-5D, Oxford Hip Score, FJS-12) were collected preoperatively, and at each follow-up were secondary.

**Results:**

At 24 months, model-based estimates showed significantly higher BMD in the Primoris group than in the Echo group in Gruen zones 3 (mean difference 0.40 g/cm², 95% confidence interval [CI] 0.26–0.53; P < 0.001), 5 (0.20 g/cm², CI 0.07–0.33; P = 0.003), and 7 (0.27 g/cm², CI 0.14–0.41; P < 0.001). These differences corresponded to relative differences of +19.1%, +9.1%, and +14.3%, respectively, compared with the Echo group. In addition, the increase in BMD from 6 weeks to 24 months was significantly greater in the Primoris group in Gruen zone 7 (mean difference in change 0.18 g/cm², CI 0.04–0.32; P = 0.01). PROMs improved similarly in both groups, with no statistically significant between-group differences.

**Conclusion:**

At 2 years, the collum-fixated Primoris stem preserved more proximal femoral bone than the Echo stem, while PROMs were comparable between groups. The observed preservation of proximal bone stock may be advantageous for long-term bone maintenance and future revision surgery.

Total hip arthroplasty (THA) remains the gold standard for treating end-stage osteoarthritis aiming primarily to relieve pain and restore function and to improve quality of life [1]. Advances in implant design and surgical technique have substantially improved implant longevity [2]. However, challenges remain in preserving femoral bone stock and mitigation stress-shielding, both of which are critical for long-term implant stability and the prevention of osteolysis and periprosthetic fractures [3].

In revision THA, the extent of femoral bone loss strongly influences procedural complexity and complication risk, including fractures and difficulties achieving stable fixation. Advanced bone loss is associated with poorer outcomes and more demanding reconstructions, underscoring the importance of bone-preserving strategies in primary THA to maintain reconstructive options for future revisions [4].

The demand for THA is increasing globally. Specifically, in the Nordic region, projections indicate a continuous rise in the volume of both primary and revision surgeries through 2050 [5]. This increasing demographic burden underscores the critical need for implant-based strategies that prioritize bone preservation and long-term stability to optimize outcomes and manage the growing need for future revision procedures.

Short-stem uncemented femoral implants have been introduced to address this need by promoting proximal load transfer while maintaining stability. Potential advantages include faster recovery, improved patient-reported outcomes, and easier revision surgery [6-8].

While evidence comparing short-stem and conventional femoral stems regarding bone remodeling and clinical outcomes has been limited, earlier findings have demonstrated that the Primoris stem provides excellent primary stability and promotes bone preservation in key proximal regions, with satisfying clinical outcomes at 5-year follow-up [9].

The aim of our study was to compare the 2 uncemented femoral stems, Primoris (short, collum-fixated) and Echo (conventional metaphyseal-fixated and diaphyseal engaging), over a 2-year period, focusing on bone preservation and clinical performance (Figure 1). The primary outcome was group-specific periprosthetic bone mineral density at 2 years, alongside between-group differences, while PROMs were assessed as secondary outcomes.

**Figure 1:**
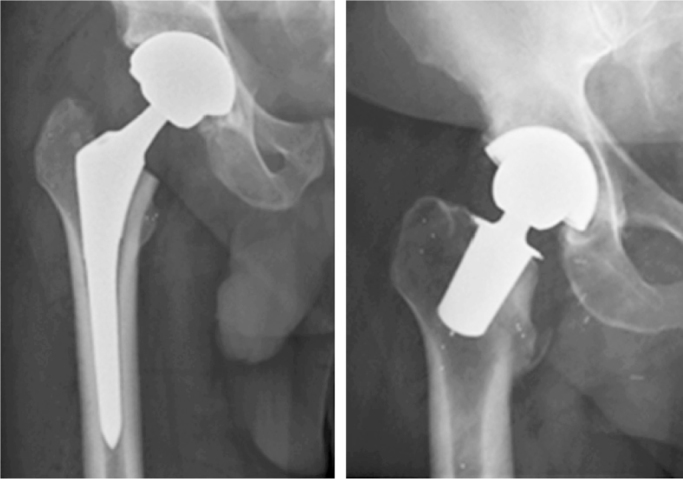
Radiographs of Echo and Primoris total hip arthroplasties.

We hypothesized that a short collum-fixated stem (Primoris) would better preserve proximal bone mineral density (BMD) than a conventional metaphyseal-fixated and diaphyseal engaging stem (Echo).

## Methods

### Study design

This randomized controlled trial (RCT) was conducted at Farsø Orthopaedic Clinic, Aalborg University Hospital, Denmark. The study adhered to the Declaration of Helsinki and was reported in compliance with CONSORT guidelines.

### Participants

Eligible patients were adults with primary osteoarthritis. Inclusion criteria specified female patients aged ≤ 55 years and male patients aged ≤ 65 years, to minimize age-related bone loss, particularly in women at higher risk of osteoporosis. Exclusion criteria included metabolic bone disease, diabetes, rheumatoid arthritis, prior hip fracture or surgery, osteoporosis (DXA T-score ≤ −2.5), current smoking, excessive alcohol consumption, and use of medications affecting bone metabolism (e.g., corticosteroids, bisphosphonates). Patients with abnormal proximal femoral anatomy or bone disease, including Legg–Calvé–Perthes disease, slipped capital femoral epiphysis, or a collum angle <125° or > 145°, were also excluded, as were patients undergoing bilateral procedures.

### Interventions

Preoperative planning was performed for both femoral stem designs in all patients. To ensure sufficient statistical power and account for potential dropout or exclusion post-randomization, recruitment was continued until 65 patients were enrolled and were screened for eligibility. Of these, 4 patients were excluded prior to randomization: 1 due to withdrawal of consent, 2 because they opted for treatment at a different facility via the patient guarantee scheme, and 1 due to incorrect selection (presence of rheumatoid arthritis). The remaining 61 patients were then randomized immediately prior to sterile unpacking of the surgical instruments, using blocks of 10 (5 Primoris and 5 Echo allocations per block). Following randomization, 1 patient in the Primoris group was excluded from the final analysis due to a periprosthetic fracture identified during the routine 6-week radiographic follow-up. Consequently, the final analyzed cohort comprised 60 patients, with 30 patients in each group.

### Variables and outcome measures

The primary outcome was the change in periprosthetic BMD, measured by DXA at 6 weeks, 12 months, and 24 months postoperatively. Secondary outcomes included patient-reported outcome measures (PROMs), including the EQ-5D, Oxford Hip Score (OHS), and Forgotten Joint Score (FJS-12), assessed preoperatively and at follow-up intervals.

### Sample size and power considerations

The sample size was calculated to detect a minimum clinically important difference (MCID) of 8% in BMD changes measured by DXA. This MCID was based on previous studies showing clinically meaningful differences in bone loss between ultra-short and conventional femoral stems [10]. With a significance level of 0.05 and 80% power, the required sample size was 25 patients per group. To account for a 10–20% dropout rate, 30 patients were recruited per group.

### Surgical procedure

All surgeries were performed by a senior orthopedic surgeon (PTN) using a standardized posterior approach. The Primoris femoral stem was positioned along the femoral neck axis, with a neck resection height approximately 25 mm proximal to the superior border of the lesser trochanter. The stem was implanted using a bone compaction technique to enhance primary stability [11]. The Echo femoral stem was implanted following proximal diaphyseal tapered reaming and using a broaching technique until the optimal stem size was achieved.

Postoperatively, patients were allowed full weightbearing as tolerated, using crutches for support during the first 4 weeks.

### Implants

The Primoris femoral stem (Zimmer Biomet, Warsaw, IN, USA), as described by Christiansen et al. [12], is a short, titanium alloy implant designed for collum-based fixation using a compaction technique with preservation of the femoral neck. The stem features a straight geometry with a length of 40–52 mm at the femoral contact area. It should be noted that the Primoris stem has since been discontinued.

In contrast, the Echo Bi-Metric femoral stem (Zimmer Biomet, Warsaw, IN, USA) is a conventional-length titanium alloy implant designed for metaphyseal and diaphyseal fixation using a broaching technique. It is a tapered wedge, non-collared, press-fit stem with proximal porous coating achieved through titanium plasma-spray and has an overall length of approximately 150 mm. This design remains commercially available.

All patients received an uncemented acetabular component (Exceed cup, Zimmer Biomet) with a liner made of vitamin-E-infused, highly cross-linked polyethylene (E1 Hi-wall liner, Zimmer Biomet) and a 36-mm cobalt-chromium-molybdenum (CoCrMo) femoral head.

### BMD measurement

BMD was measured in g/cm², using DXA with a Norland XR-36 densitometer (Norland Corp, Fort Atkinson, WI, USA). Preoperative DXA of the hip and lumbar spine was used to exclude patients with osteoporosis (T-score ≤ −2.5) (see Table 1).

**Table 1:** Preoperative baseline data

Item	Primoris n = 31	Echo n = 30
Demographics		
Male / female	26/5	24/6
Mean age (SD)	54 (8.1)	54 (6.1)
Mean height, cm (SD)	180 (7.3)	178 (6.8)
Mean weight, kg (SD)	91 (12)	85 (10)
Mean BMI (SD)	28 (2.9)	27 (3.0)
Preoperative DXA scan		
Completion rate	27	26
Femoral neck BMD g/cm^2^ (SD)	1.00 (0.16)	0.93 (0.13)
Femoral trochanter BMD g/cm^2^ (SD)	0.85 (0.15)	0.82 (0.13)
Femoral T-score (SD)	–0.006 (1.3)	–0.52 (1.2)
L2–4 BMD g/cm^2^ (SD)	1.2 (0.17)	1.1 (0.16)
L2–4 T-score (SD)	0.51 (1.5)	0.06 (1.4)

BMD: bone mineral density; BMI: body mass index; SD: standard deviation.

Postoperative DXA scans were performed on the operated-on hip at 6 weeks, 12 months, and 24 months. Due to the collum-positioned Primoris stem, the conventional 7 Gruen zones were modified. Instead of being defined by the implant geometry, the zones were anatomically defined based on the native proximal femur to allow for consistent and meaningful comparison of regional BMD changes (see Figure 3).

### Patient-reported outcome measures (PROMs)

We compared the PROMs, European Quality of life (EQ-5D), Oxford Hip Score (OHS), Forgotten Joint Score (FJS-12), preoperatively, and at 6 weeks, 1 and 2 years postoperatively.

Using all 3 instruments allows us to assess the overall health and quality of life, hip-specific pain and function, and joint awareness.

### Statistics

Bone quality was analyzed using a linear mixed-effects model (LMM) for repeated measurements. Time, prosthesis type, and region were included as fixed effects together with all interaction terms. To account for within-subject correlation due to repeated measurements, patient ID was included as a random intercept. Estimated marginal means at 24 months were obtained using the emmeans package in R (R Foundation for Statistical Computing, Vienna, Austria), and pairwise comparisons between groups were performed. Results are presented as estimated differences with 95% confidence intervals (CI) and P values.

Statistical calculations were performed using Microsoft Excel (Microsoft Corp, Redmond, WA, USA) and R (version 4.4.2).

### Ethics, registration, funding, and disclosures

The study was approved by the Regional Committee on Health Research Ethics (approval no. N-20170030). The study was registered at ClinicalTrials.gov (identifier: NCT03279276). All study data was prospectively collected and archived by dedicated research nurses at the Orthopaedic Department in Farsø. The digitized datasets are stored on a secure, access-controlled institutional research server. All participants provided written informed consent prior to inclusion.

No external funding was received. The study was internally funded by the Research Unit at Farsø, Aalborg University Hospital. All patient recruitment, surgeries, and postoperative care were part of the routine clinical activity at the hospital. There were no financial conflicts of interest. Complete disclosure of interest forms according to ICMJE are available on the article page, doi: 10.2340/17453674.2026.46481

## Results

**Figure 2:**
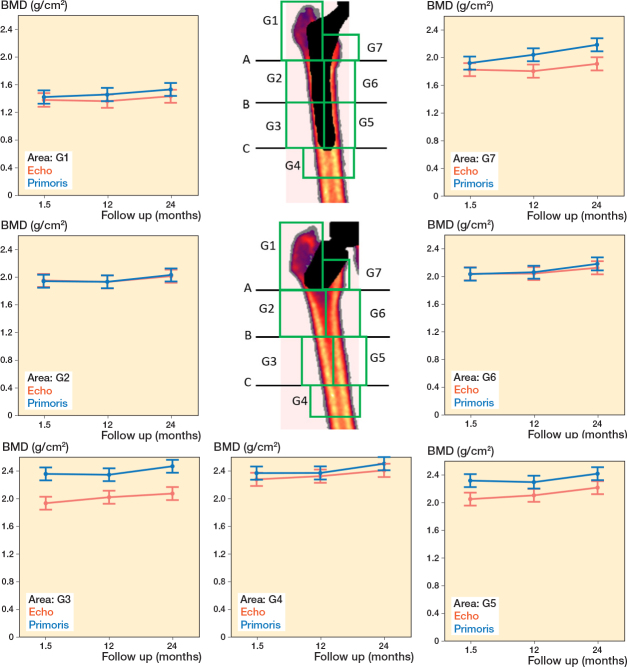
Flowchart. DXA = Dual energy X-ray Absorptiometry.

Between April 2018 and October 2019, 954 patients scheduled for primary total hip arthroplasty (THA) were screened, of whom 61 fulfilled the inclusion criteria and were deemed suitable candidates for both stem designs (Figure 2). Postoperative BMD was assessed in 7 anatomical Gruen zones at 6 weeks, 12 months, and 24 months, yielding 630 data points per group. Outliers, defined as > 3 SD from the mean, were excluded (7/630 in the Primoris group and 10/630 in the Echo group). These outliers were predominantly located in Gruen zone 1 and were attributed to artifactual BMD elevation caused by the femoral stem. The overall proportion of missing data was 3.3% in the Primoris group and 6.0% in the Echo group.

Preoperative DXA scans revealed similar bone mineral density in the 2 groups at the femoral trochanteric region, femoral neck, or lumbar spine (L2–L4). The completion rate was 87% in both groups (Table 1).

At 2 years, the lowest estimated BMD was observed in Gruen zone 1: 1.53 g/cm² (CI 1.44–1.63) in the Primoris group and 1.43 g/cm² (CI 1.34–1.53) in the Echo group. The highest BMD was in Gruen zone 4: 2.51 g/cm² (CI 2.41–2.60) and 2.41 g/cm² (CI 2.32–2.51), respectively.

### Primary outcome

At 2 years postoperatively, between-group comparisons demonstrated higher BMD in the Primoris group in Gruen zone 3 (mean difference 0.40 g/cm², CI 0.26–0.53; P < 0.001), zone 5 (0.20 g/cm², CI 0.07–0.33; P = 0.003), and zone 7 (0.27 g/cm², CI 0.14–0.41; P < 0.001). In addition, the increase in BMD from 6 weeks to 24 months was significantly greater in the Primoris group in Gruen zone 7 (mean difference in change 0.18 g/cm², CI 0.04–0.32; P = 0.01) (Table 2).

**Table 2:** Postoperative DXA score for the short Primoris and conventional Echo stem. BMD reported as g/cm^2^

Anatomical Gruen zone (G1–7)		Primoris n = 30	Echo n = 30	Echo vs Primoris change/difference (CI)	P value
G1	Observed mean BMD (SD), n				
	6 weeks	1.47 (0.17), 26	1.44 (0.19), 24		
	12 months	1.51 (0.19), 26	1.40 (0.16), 26		
	24 months	1.54 (0.16), 29	1.43 (0.19), 28		
	Change 6 weeks to 24 months				
	Relative BMD% (SD) **^a^**	6.2 (12.7)	–0.9 (7.2)		
	EMM BMD change (CI) **^b^**			0.06 (–0.08 to 0.20)	0.4
	At 24 months				
	EMM BMD (CI) **^b^**	1.53 (1.44–1.63)	1.44 (1.34–1.53)	Relative diff. **^c^**: 6.9%	
	EMM BMD difference (CI) **^b^**			0.10 (–0.03 to 0.23)	0.1
G2	Observed mean BMD (SD), n				
	6 weeks	1.95 (0.29), 29	1.96 (0.27), 29		
	12 months	1.93 (0.30), 30	1.93 (0.31), 29		
	24 months	2.04 (0.18), 29	2.01 (0.24), 28		
	Change 6 weeks to 24 months				
	Relative BMD% (SD) **^a^**	6.0 (15.4)	4.7 (18.2)		
	EMM BMD change (CI) **^b^**			0.02 (–0.12 to 0.16)	0.7
	At 24 months				
	EMM BMD (CI) **^b^**	2.03 (1.94–2.13)	2.01 (1.92–2.11)	Relative diff. **^c^**: 0.8%	
	EMM BMD difference (CI) **^b^**			0.02 (–0.12 to 0.15)	0.8
G3	Observed mean BMD (SD), n				
	6 weeks	2.36 (0.26), 29	1.94 (0.35), 29		
	12 months	2.35 (0.25), 30	2.03 (0.32), 28		
	24 months	2.48 (0.15), 29	2.07 (0.25), 28		
	Change 6 weeks to 24 months				
	Relative BMD% (SD) **^a^**	5.2 (11.9)	10.8 (22.2)		
	EMM BMD change (CI) **^b^**			–0.06 (–0.20 to 0.09)	0.4
	At 24 months				
	EMM BMD (CI) b	2.47 (2.37–2.56)	2.07 (1.98–2.17)	Relative diff. **^c^**: 19.1%	
	EMM BMD difference (CI) **^b^**			0.40 (0.26 to 0.53)	< 0.001
G4	Observed mean BMD (SD), n				
	6 weeks	2.38 (0.25), 29	2.29 (0.20), 29		
	12 months	2.38 (0.28), 30	2.33 (0.25), 29		
	24 months	2.51 (0.18), 29	2.41 (0.18), 28		
	Change 6 weeks to 24 months				
	Relative BMD% (SD) **^a^**	6.4 (10.5)	5.9 (11.5)		
	EMM BMD change (CI) **^b^**			0.02 (–0.13 to 0.16)	0.8
	At 24 months				
	EMM BMD (CI) **^b^**	2.51 (2.41–2.60)	2.41 (2.32–2.51)	Relative diff. **^c^**: 3.9%	
	EMM BMD difference (CI) **^b^**			0.10 (–0.04 to 0.23)	0.2
G5	Observed mean BMD (SD), n				
	6 weeks	2.33 (0.26), 29	2.06 (0.25), 29		
	12 months	2.30 (0.28), 30	2.11 (0.32), 29		
	24 months	2.43 (0.18), 29	2.21 (0.19), 28		
	Change 6 weeks to 24 months				
	Relative BMD% (SD) **^a^**	5.3 (14.4)	9.2 (15.1)		
	EMM BMD change (CI) **^b^**			–0.07 (–0.21 to 0.07)	0.3
	At 24 months				
	EMM BMD (CI) **^b^**	2.42 (2.33–2.51)	2.22 (2.12– 2.31)	Relative diff. **^c^**: 9.1%	
	EMM BMD difference (CI) **^b^**			0.20 (0.07 to 0.33)	0.003
G6	Observed mean BMD (SD), n				
	6 weeks	2.04 (0.27), 29	2.05 (0.29), 29		
	12 months	2.06 (0.29), 30	2.05 (0.30), 29		
	24 months	2.19 (0.19), 29	2.12 (0.26), 28		
	Change 6 weeks to 24 months				
	Relative BMD% (SD) **^a^**	8.2 (14.7)	5.3 (17.7)		
	EMM BMD change (CI) **^b^**			0.07 (–0.08 to 0.21)	0.4
	At 24 months				
	EMM BMD (CI) **^b^**	2.18 (2.09–2.28)	2.13 (2.03–2.22)	Relative diff. **^c^**: 2.6%	
	EMM BMD difference (CI) b			0.06 (–0.08 to 0.19)	0. 3
G7	Observed mean BMD (SD), n				
	6 weeks	1.93 (0.28), 29	1.83 (0.35), 29		
	12 months	2.04 (0.32), 30	1.82 (0.33), 28		
	24 months	2.19 (0.21), 29	1.90 (0.33), 28		
	Change 6 weeks to 24 months				
	Relative BMD% (SD) **^a^**	14.7 (19.0)	5.9 (22.5)		
	EMM BMD change (CI) **^b^**			0.18 (0.04 to 0.32)	0.01
	At 24 months				
	EMM BMD (CI) **^b^**	2.18 (2.09–2.28)	1.91 (1.82–2.01)	Relative diff. **^c^**: 14.3%	
	EMM BMD difference (CI) **^b^**			0.27 (0.14 to 0.41)	< 0.001

Foot note for Table 2.

EMM = estimated marginal mean; SD = standard deviation; n = number of patients in each group for whom accurate BMD measurements were obtained.

a Descriptive percentage change calculated from individual patient values.

b Linear mixed-effects model (LMM) for repeated measurements.

c Relative differences were calculated from estimated marginal means at 24 months using

Echo as reference.

**Figure 3:**
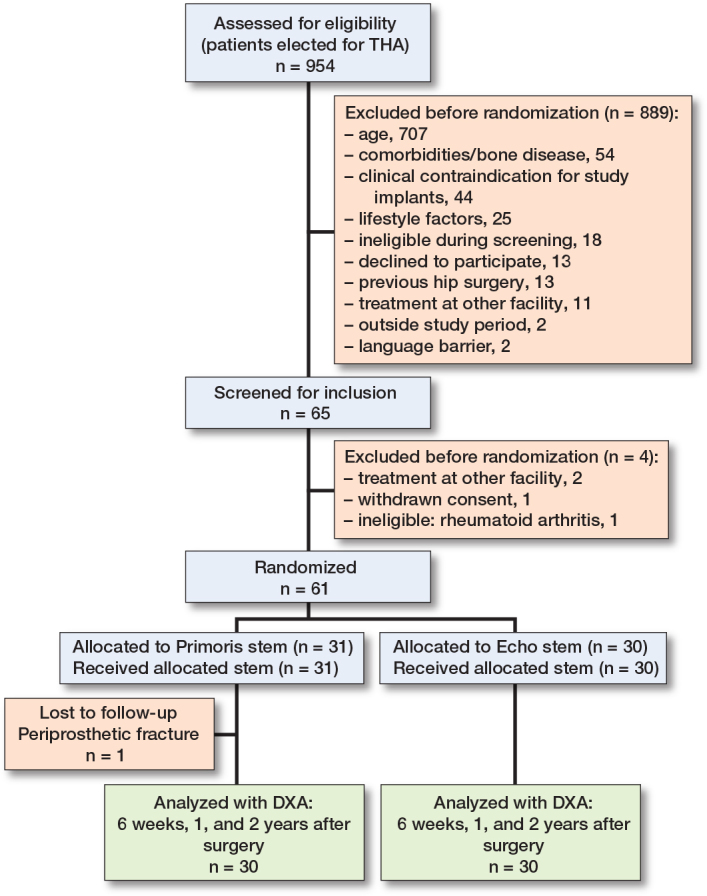
Anatomical Gruen zones and BMD over time by Gruen zone with the Primoris or Echo prothesis. Data is presented as estimated marginal means with 95% confidence intervals. Throughout this article, all references to Gruen zones refer to the anatomically defined Gruen zones described below. Anatomically defined Gruen zones 1–7: The zones are defined relative to the center of the femoral shaft and numbered counterclockwise, corresponding to the conventional Gruen classification. The horizontal lines (A, B, and C) define the proximal and distal boundaries of the zones. Line A: Horizontal line centered at the medial apex of the lesser trochanter. Line B: Horizontal line 5 cm distal to line A. Line C: Horizontal line 10 cm distal to line A.

Postoperative changes in periprosthetic estimated BMD in Gruen zones 1–7 are shown in Figure 3 at 6 weeks, 1 year, and 2 years postoperatively.

### PROM results

At 2 years postoperatively, observed (raw) PROM scores were similar for the Primoris and Echo stems. The adjusted between-group differences at 2 years were 0.046 (CI –0.039 to 0.131; P = 0.3) for EQ-5D index and 3.7 points (CI –9.3 to 17.8; P = 0.6) for EQ VAS. EQ-5D completion rates were 100% preoperatively, 98% at 6 weeks, 97% at 1 year, and 97% at 2 years.

The adjusted OHS between-group difference at 2 years was –2.1 points (CI –8.6 to 4.4; P = 0.5). OHS ranges from 12 (best) to 60 (worst), and completion rates were 100% preoperatively, 98% at 6 weeks and 1 year, and 97% at 2 years.

The adjusted FJS between-group difference at 2 years was 8.0 points (CI –12.5 to 28.5; P = 0.5). FJS ranges from 0 (worst) to 100 (best), with completion rates of 95% at 6 weeks, 97% at 1 year, and 96% at 2 years.

**Figure 4:**
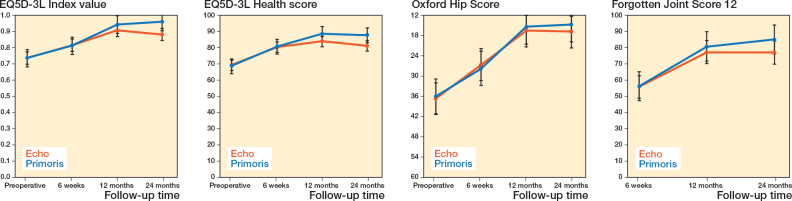
PROM scores by follow-up time. EQ5D-3L: A health-related quality of life instrument consisting of 2 components: 1) Index, a multidimensional scale assessing mobility, self-care, usual activities, pain, and anxiety; and 2) Health score: a visual analogue scale (VAS) representing the patient’s self-evaluated health status (0–100, higher = better). Oxford hip score (OHS): A patient-reported outcome measure assessing functional status and hip-related pain (60–12, lower = better). Forgotten Joint Score 12 (FJS12): A patient-reported outcome measure assessing the degree of joint awareness and the ability to perform daily activities without conscious thought of the prosthesis (0–100, higher = better).

No statistically significant differences were observed between groups for any PROM at any time point (Figure 4).

## Discussion

We aimed to evaluate the effectiveness of 2 uncemented femoral stems, Primoris (short, collum-fixated) and Echo (conventional metaphyseal-fixated and diaphyseal engaging), and found that the collum-fixated, non-diaphyseal engaging stem demonstrated enhanced bone preservation in select Gruen zones, compared with a conventional stem design. This effect is partially attributable to the different bone preparation techniques: the collum-fixated stem utilizes a bone compaction approach within a relatively limited bone area prior to implantation.

Longitudinal evaluation suggests that the diaphyseal-sparring design may facilitate more physiological load transfer, potentially conferring enhanced bone preservation benefits in selected patient cohorts. This hypothesis is supported by the observed increase in BMD within Gruen zone 7 from 6 weeks to 2 years, representing the most substantial increase in BMD between the 2 stem types (collum-fixated: +14.7%; conventional +5.9%). At 2 years, the collum-fixated stem exhibited significantly higher estimated BMD in Gruen zone 7, at 0.18 g/cm2 (CI 0.04–0.32) P = 0.01.

This enhanced bone preservation is consistent with the stable biomechanical profile of the Primoris stem. Although implant migration can theoretically trigger mechanobiological remodeling, previous RSA data for this stem demonstrated excellent stability, with minimal subsidence (mean 0.38 mm) and varus tilt (mean 0.93°) [12]. Furthermore, that study established a significant correlation between reduced implant motion and increased BMD [12], suggesting that the bone remodeling observed in our cohort is driven by stable, optimized proximal load transfer rather than a compensatory response to implant instability. Our findings, therefore, reinforce the hypothesis that the design facilitates physiological loading.

Collectively, these findings indicate a trend toward increased proximal femoral BMD with the collum-fixated stem, which may positively influence the feasibility and outcomes of future revision procedures.

The current findings contribute valuable insights into the biomechanical behavior of collum-fixated femoral stems compared with longer metaphyseal- and diaphyseal-engaging designs and contribute to the ongoing development and optimization of short-stem prostheses. The Primoris stem is no longer in production, but corresponds to a Type 1A design under the Khanuja et al. [13] classification, featuring femoral neck-only fixation and a trapezoidal cross-section.

Peak bone ingrowth and BMD stabilization following implantation of porous-coated, cementless femoral stems typically occur between 12 and 24 months [14]. As stem design dictates bone loading patterns and subsequent remodeling according to Wolff’s law, variations in BMD response are to be expected.

MCID for changes in periprosthetic BMD has been estimated at 8% [10]. The observed increase in zone 7 BMD in the Primoris group was 14.7%, exceeding this threshold and suggesting a potentially clinically relevant effect within the 2-year follow-up period. Preservation or improvement of proximal femoral bone stock may be advantageous in the event of future revision surgery. However, this finding should be interpreted with caution, as the percentage change was derived from descriptive analyses based on individual patient values.

In the greater trochanter (AG zone 1), no between-group difference in BMD was observed, and both stem types showed the lowest BMD in this region. Similarly, no differences were found in AG zones 2 and 6. In the diaphyseal regions (AG zones 3 and 5), BMD was generally higher in the Primoris group, with between-group differences at 24 months. These findings may reflect differences in surgical impact on the proximal femur, with the Primoris stem potentially preserving diaphyseal bone to a greater extent than the Echo stem. In the distal diaphyseal region (AG zone 4), BMD was highest for both stem types, and no between-group difference was observed

### Limitations

The study’s external validity is limited by selective inclusion criteria, potentially introducing sex-related bias with the limited number of female participants.

While long-term data would provide further insight into stress shielding and bone remodeling, Nielsen et al. [8] reported a 7.8% decrease in proximal femoral BMD over 10 years following Primoris stem implantation, potentially reflecting age-related bone loss.

DXA measurements in localized femoral zones (e.g., Gruen zone 7) are susceptible to low reproducibility and positioning errors, potentially impacting BMD accuracy, particularly in the presence of metallic implants.

This study adapted standard Gruen zones for femoral BMD assessment across implants with differing designs. While intending regional comparisons, this approach resulted in evaluating periprosthetic bone in one group vs non-periprosthetic bone in another, potentially influencing cortical-to-trabecular bone ratios. However, significantly higher BMD was observed in Gruen zones 3 and 5 at 6 weeks post-THA in the short stem group. This suggests that cortical bone predominantly contributes to BMD in the Gruen zones, and the impact of trabecular bone elimination by the implant stem was not readily detectable in early measurements. Caution is warranted when interpreting early post-THA BMD, as surgical trauma and bone compaction may confound accurate assessment of long-term bone quality; longitudinal follow-up, such as 2-year BMD data, is crucial for evaluating implant impact.

### Conclusions

We showed that the ultra-short collum-fixated stem had higher BMD than the conventional metaphyseal-fixated stem (Echo) at 2 years in Gruen zones 3, 5, and 7. Both stems provided favorable and similar patient-reported outcomes. These findings indicate that neck-preserving short stems may support physiological load transfer and proximal bone preservation, although longer-term results remain to be established.

## Notes

AT: formal analysis, investigation, methodology, project administration, and writing—original draft. JDC: formal analysis. TJ: project administration and supervision. PTN: conceptualization, methodology, project administration, supervision, and writing—review and editing.

The authors thank Gitte B. Hansen and Merete Hessellund for nursing assistance and Martin Jensen for statistical support.

Handling co-editors: Keijo Mäkelä and Robin Christensen

*Acta* thanks Harald Brismar and Reinhard Windhager for help with peer review of this manuscript.
